# Enhancing Wastewater Treatment Efficiency: Utilising Saponification Products for Sustainable Cleaning Processes

**DOI:** 10.1111/1758-2229.70124

**Published:** 2025-07-09

**Authors:** Dani Dordević, Monika Vítězová, Tomáš Vítěz, Simona Dordevic, Monika Hamšíková, Ivan Kushkevych

**Affiliations:** ^1^ Department of Plant Origin Food Sciences Faculty of Veterinary Hygiene and Ecology, University of Veterinary Sciences Brno Brno Czech Republic; ^2^ Department of Experimental Biology Faculty of Science, Masaryk University Brno Czech Republic; ^3^ Department of Agricultural, Food and Environmental Engineering, Faculty of AgriSciences Mendel University in Brno Brno Czech Republic

**Keywords:** edible oil waste, microorganism respiration, sustainability, wastewater treatment efficiency

## Abstract

This study explores the interaction of saponification products with microbial communities in aerobic and anaerobic sewage sludge from wastewater treatment plants (WWTPs). It focuses on the reutilisation of waste cooking oils into soap and evaluates the biodegradation of these products using microbial respiration activity and biological oxygen demand (BOD) as indicators. Results demonstrate that soaps degrade effectively under both aerobic and anaerobic conditions, with anaerobic degradation contributing to methane production—a valuable biofuel. Importantly, no toxic effects on sludge microorganisms were observed. The research highlights that these saponification products can be fully integrated into the wastewater treatment process without adverse effects on microbial dynamics. Moreover, the economic analysis reveals that biosurfactants derived from used oils can be produced at a cost of approximately 0.12–3.0 EUR/kg, significantly lower than the 1–20 EUR/kg typically spent on chemical coagulants or synthetic surfactants used in WWTPs. These findings support the feasibility of repurposing waste oils into environmentally friendly, cost‐effective treatment additives, enhancing microbial performance and promoting circular economy practices in wastewater management.

## Graphical abstract

This study demonstrates the successful utilisation of saponification products derived from used cooking oils to enhance wastewater treatment efficiency. These products support microbial respiration and biofuel production, offering a sustainable approach to waste management and resource recovery within wastewater treatment systems.

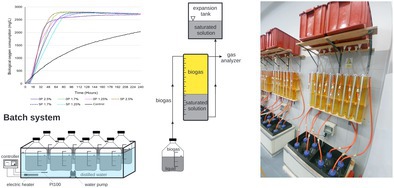

## 1 Introduction

In 2022, the human population has reached 8 billion people. It is certain that with the growing population and human activities, the amount of waste produced will also increase (Adam 2021). Global impacts can also be observed in the increasing production of raw materials, especially primary crops such as sugar cane, cereals, vegetables and also oilseeds. According to the report from the Food and Agriculture Organisation of the United Nations (FAO), global oilseed production for the 2023/24 season is projected to hit a record 666.7 million tons. The production of oilseeds recorded the highest increase between 2000 and 2020, namely by 120% from 0.5 billion to 1.1 billion. The Food and Agriculture Organisation (FAO) estimates the production of oilseeds between 2020 and 2021 at a total of 616.4 billion tonnes, of which 242.9 billion tonnes are vegetable oils and fats. The production of palm oil dominates, with two countries—India and Malaysia—accounting for 85% of this (FAO 2022; Jarvis 2023).

Vegetable cooking oil is commonly used for frying, cooking or baking in households, restaurants, hotels and food services. During frying, the oil undergoes a series of physical and chemical changes in the structure of the triacylglycerols at high temperatures (160°C–200°C). In addition, reactions such as oxidation, hydrolysis and polymerisation produce toxic compounds that have harmful effects on human health (Awogbemi et al. 2021).

The issue of using used cooking oil is becoming increasingly important with regard to the environment. One litre of oil released into the environment can pollute up to 500,000 L of water. Oil increases the pollution of water bodies with organic substances and forms a thin layer on the surface that reduces access to oxygen for aquatic organisms. Cooking oil poured directly into the sink can solidify and block the drain pipe. The problem also occurs in wastewater treatment plants, where operating costs for oil removal increase (Panadare and Rathod 2015). Used waste oil is a cost‐effective raw material that can be used for other purposes. For example, one of the sustainable options is to add cooking oil to materials such as bitumen or asphalt to regenerate worn roads, same as to serve as the renewable fuel source in the production of biodiesel (Luo and Liu 2023; Shrivastava et al. 2023; Zahoor et al. 2021).

Another option is the use of used cooking oils in the form of saponification products. Their production in the form of soap is simple and inexpensive, and their degradation is made possible by microorganisms, especially in biological wastewater treatment. One example of this is anaerobic microorganisms such as *Synthrophomonas* sp., an anaerobic, obligate syntrophic, fatty acid‐degrading acetogenic bacterium for which soap can be an ideal carbon source (Antonić et al. 2020).

The soap and oil industries may generate relatively small amounts of liquid waste directly, but they are a source of great public concern when their products enter nearby waterways. These residues are known to cause several problems, including: (1) disruption of oxygen transfer in activated sludge treatment and receiving streams, (2) excessive foaming, (3) toxicity to freshwater fish and (4) difficult removal at water treatment plants (Abdel‐Gawad and Abdel‐Shafy 2002).

Both aerobic and anaerobic processes are used in the biological treatment of wastewater. The use of soaps made from cooking oil brings the potential for the utilisation of this oil and the prevention of problems that occur at sewage treatment plants. Degradation of these soaps using microbial metabolism (aerobic respiration) is often represented by respirometric biological oxygen demand (BOD). By analysing the mixed culture of activated sludge and anaerobic sludge used in wastewater treatment, it is possible to determine the biological activity and influence of the produced soaps on the cleaning process.

The saponification products become part of the wastewater that flows through the wastewater treatment plant. There they influence the microbial activity of the sewage sludge. The aim of the study is to find out what influence different saponification products have on the respiration activity of activated sludge in the aerobic phase of wastewater treatment and anaerobic stabilisation. The respiration activity is an important indicator of the biological oxygen consumption at a certain temperature and for a certain time and expresses the state of the microbial population in the activated sludge, which is influenced by the addition of saponification products. The aim of the study was to test the three most commonly used edible oils—palm, rapeseed and sunflower oil—with different degrees of oil burnout. The production of biogas and methane from the anaerobic stabilisation of sewage sludge was determined, and the possible influence of saponification products on this process was evaluated.

## 2 Material and Methods

### 2.1 Experimentally Produced Soaps

The soaps were produced at the University of Veterinary Sciences Brno in the Czech Republic. For the experimental purposes of soap production, palm oil (packaged in Austria) was selected as the most commonly used edible oil. French fries (Hearty Food Co., Tesco, Prague, Czech Republic) were used to simulate the regular process of deep‐frying. French fries (100 g) were fried in 3.3 L of oil in a deep fryer (CONCEPT FR 2035) for 5 min at a temperature of 175°C. Using the Dęsto 270 device (Dęsto SE & Co. KGaA, Germany), the content of total polar compounds (CPH, from TPC–) was measured at the following five levels: 5%, 10%, 15%, 20% and 24% (Table 1). The oil samples obtained were used as material for the production of soaps in the cold saponification process. Fresh oils were used as control samples. The samples were labelled according to the degree of burnout and the type of oil used (Table 1).

**TABLE 1:** Determining the degree of total polar matter in experimentally produced soap samples.

Sample	Degree of burnout for P, R, S*	Total polar matter content (%)
Control	0	5
Sample 1 sets	1	6.5
Sample 2 sets	2	10
Sample 3 sets	3	15
Sample 4 sets	4	20
Sample 5 sets	5	24

* Palm oil (P), Rapeseed oil, Sunflower oil (S).

The soaps were produced using a cold saponification process in the laboratory, in which the oil was continuously heated to 200°C and cooled at room temperature for 20 min. Three cycles of heating and cooling were repeated. The recipe for soap production was as follows: 130 g of oil, 17.52 g of NaOH and 49.40 g of distilled water were used for the process. The aqueous NaOH solution was first cooled to room temperature and then mixed with the oil using a mixer until a pudding‐like consistency was achieved. The mixture was then poured into a silicone mould and left to harden for 24 h. After solidification, the solidified mixture was placed on filter paper and allowed to mature for 4 weeks (Antonić et al. 2020).

In an earlier experiment, the oil was tested for acidity, hydrogen peroxide and malondialdehyde (MDA), while the soap samples were analysed for various pH values, solids content, alkalinity, fat content, MDA, strength and brittleness. Among the most significant differences between the parameters was the malondialdehyde content—oils that received 24% CPH in the final stage of frying contained up to 6.56 μg/g (Antonic et al. 2021). Oils with long chains of unsaturated fatty acids are more susceptible to heating and thus to increased formation of MDA as an end product of lipid peroxidation. In addition to the chain length, the additives used for frying, which contain transition metals, also influence peroxidation. Free transition metal ions (e.g., Ni, Fe, Cu) react with hydrogen peroxide and form highly reactive radicals and MDA (Doureradjou and Koner 2008).

### 2.2 Basic Analysis

The dry matter (DM) content was determined by drying the samples at 105°C ± 2°C to a constant weight and then cooling the samples in a desiccator according to the Czech standard method ČSN EN 15934 (838125). The content of organic dry matter (ODM) was determined by the incineration of samples in a muffle furnace at 550°C ± 5°C according to the Czech standard method (ČSN EN 15935 2013). An LMH 04/12 muffle furnace (LAC Ltd., Czech Republic) was used for the analysis. pH and redox potential were measured with a GHM 5500 multimeter (Greisinger, Germany) and conductivity with a WTW 3320 multimeter (WTW Xylem Analytics, Germany).

### 2.3 Measurement of Respiratory Activity

**FIGURE 1:**
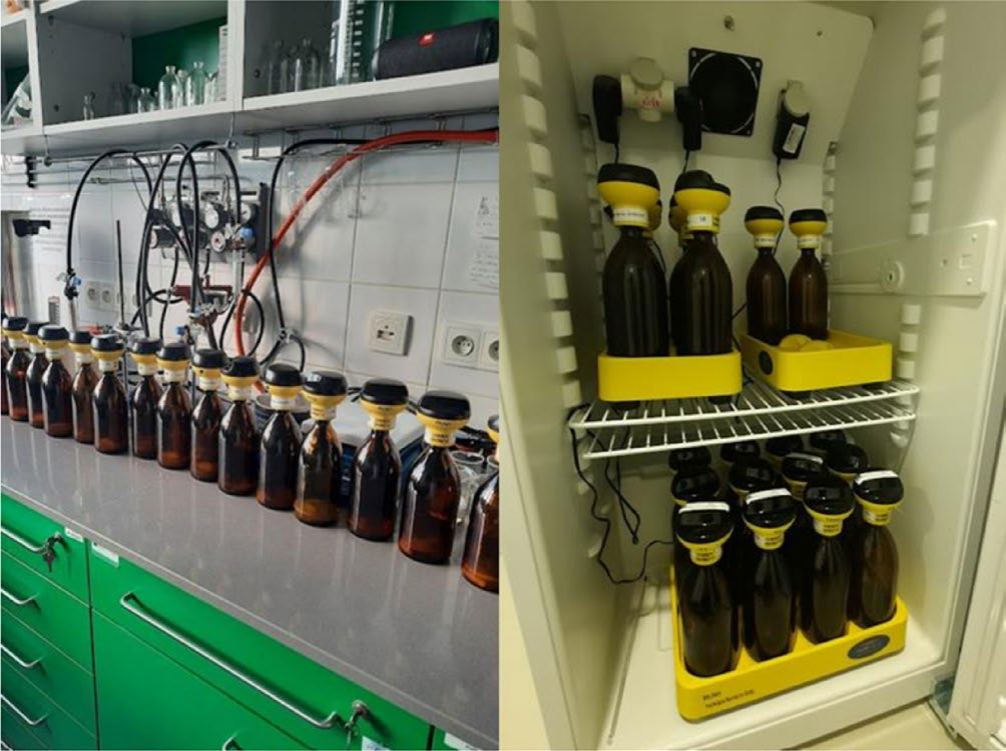
OxiTop experiment set‐up (author).

An OxiTop system with OC 110 controller (Figure 1) (WTW Xylem Analytics, Germany) was used to determine the biological oxygen demand (BOD) during the aerobic degradation of soap. The OxiTop system consists of dark glass bottles with a volume of 500 mL, which suppress the access of light and serve as a container for the incubated sample. The most important part is the plastic measuring head (OxiTop‐C), which contains a pressure sensor. The head continuously measures the negative pressure created by the respiration of the microorganisms in a closed bottle at a constant temperature of 20°C over a period of 5 days. The head recorded pressure values in the range of 500–1350 hPa (maximum 2000 hPa) at half‐hour intervals and converted them into a BOD value according to the equation of state (WTW 2014).

The bottles containing the inoculum, the mineral medium, the soap sample and the magnetic stirrer were fitted with rubber sleeves into which NaOH granules were inserted for CO_2_ sorption. The head of the OxiTop system was screwed tightly onto the bottle. The bottles were then synchronised with the controller of the OxiTop system. The following parameters were set: BOD standard, 10 days’ cultivation, total sample volume in the bottle 43.5 mL, and the sample identification number was selected. The bottles were then placed on magnetic stirrers in an incubator at a temperature of 20°C. At the end of the experiment, the data were read from the control unit and downloaded to the computer using the Achat OC V320 software programme.

### 2.4 Mineral Medium

A mineral medium prepared according to the official OECD/OCDE guidelines (301 A DOC test) was used for the BOD test (OECD 1992).

### 2.5 Inoculum Collection

**FIGURE 2:**
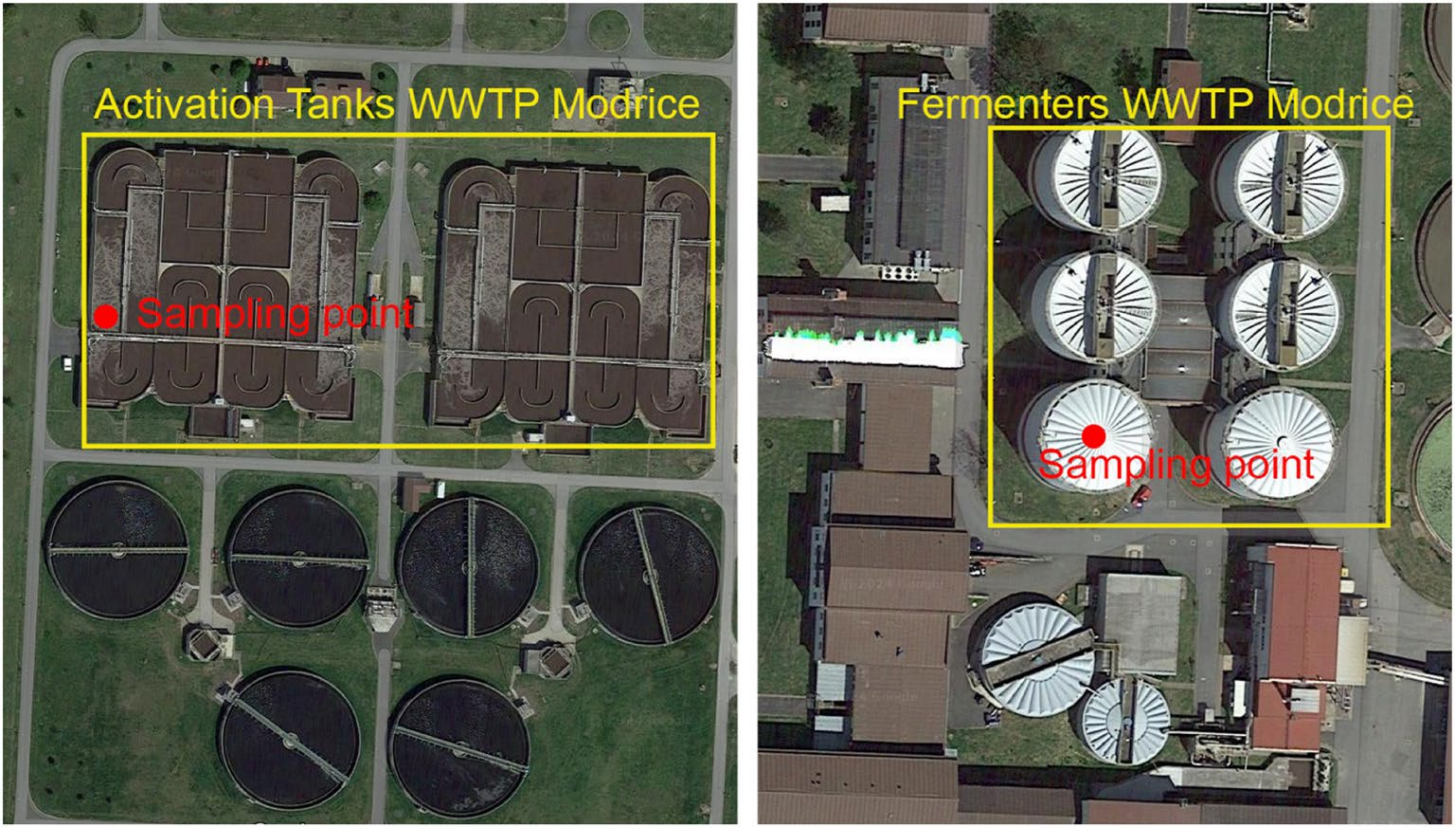
Activated sludge sampling site (left) and anaerobic sludge sampling site (right), Brno–Modřice WWTP, (edited by the author, Google maps).

The activated sludge was collected from the activation tank of the Brno—Modřice wastewater treatment plant (Czech Republic) in a plastic container with a volume of 1 L. Sampling was performed from line 4, where the nitrification zone is located (Figure 2, left). At each sampling, information was obtained on the suspended solids (SS, g/L) content in the sludge, which was measured by the operator of the wastewater treatment plant. The anaerobic sludge was taken from the anaerobic sludge stabilisation fermenter of the Brno—Modřice WWTP, which is operated in the mesophilic temperature regime at a temperature of 40°C (Figure 2, right).

### 2.6 Experiment Description

For the preparation of the inoculum, falcons were prepared with resuspended activated sludge with a volume of 30 mL. The sludge was centrifuged at 5000 rpm and 20°C for 5 min. The supernatant was then discarded, and the pellet was washed with mineral medium (see Paragraph 2.4) to a total volume of 30 mL and resuspended by vortexing. After a further centrifugation, the supernatant was again discarded, but the pellet was washed with 15 mL of mineral medium. The pellet was shaken again in the medium, and the resulting 15 mL inoculum was placed in a glass bottle with an OxiTop device to measure respiratory activity. To 15 mL of the washed activated sludge, 23.5 mL of mineral medium and 5 mL of a soap sample with different concentrations and different degrees of combustion were added.

The soap solution was prepared by dissolving a solid soap sample in the required amount of water in a beaker and heating it on an electromagnetic hotplate at a temperature of approx. 60°C while stirring. The following three solutions were prepared at a concentration of 2.5% (1 g soap in 40 mL H_2_O), 1.7% (1 g soap in 59 mL H_2_O) and 1.25% (1 g soap in 80 mL H_2_O). For each degree of initial oil burnout, 5 mL of the soap solution was inoculated into the activated sludge at a volume of 15 mL and into the mineral medium at a volume of 23.5 mL in a BOD bottle, and the respiration activity was measured. The total volume of inoculum was 43.5 mL, which corresponds to a BOD of 2000 mg/L. Each concentration at a given oil burnout degree was tested in duplicate, and these values were then averaged. Two controls containing only 15 mL of activated sludge with 28.5 mL of mineral medium without added soap were prepared simultaneously (Table S1).

### 2.7 Anaerobic Degradation

**FIGURE 3:**
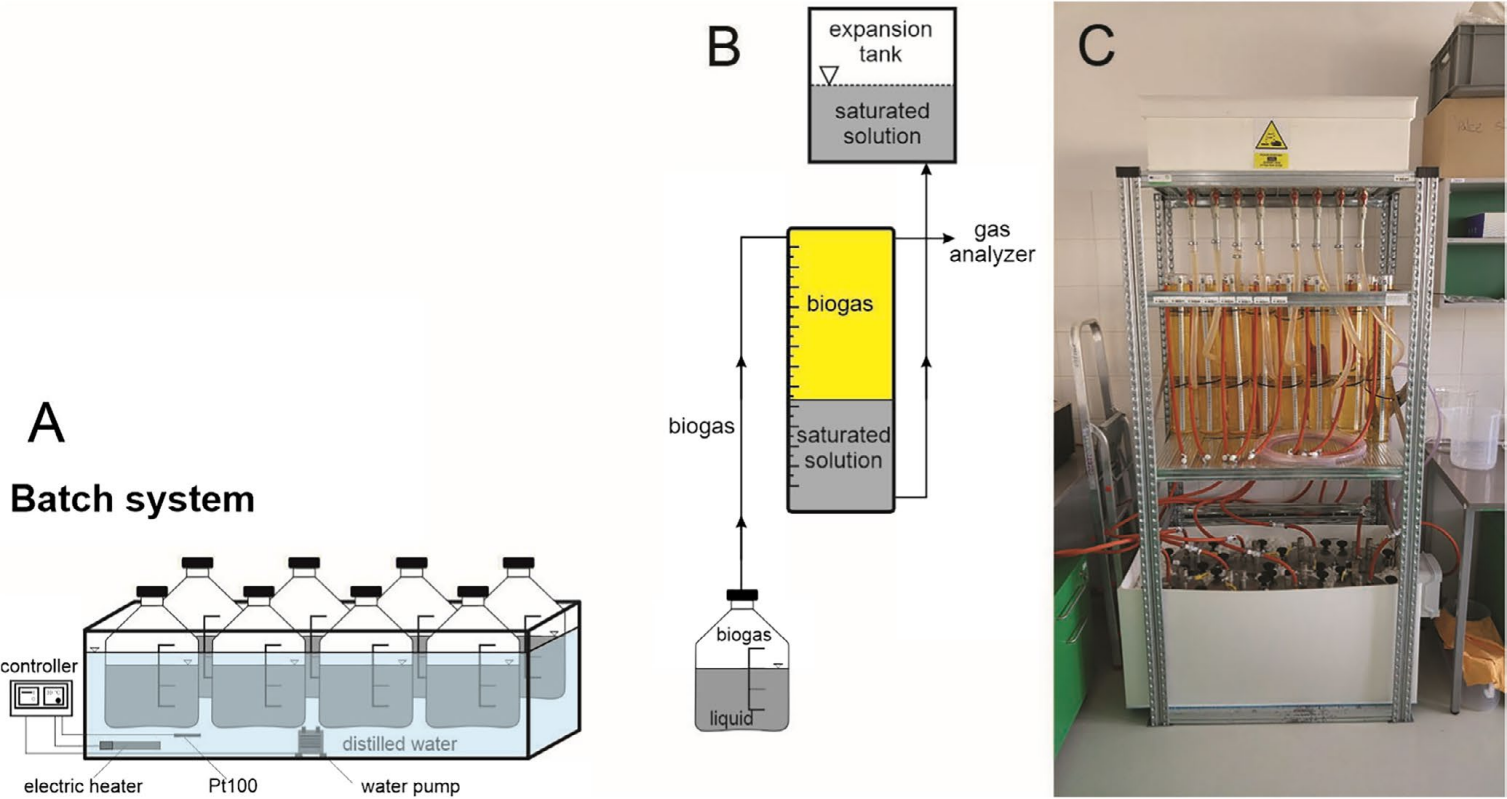
Schematic illustration of an anaerobic fermenter (Vítěz, modified by the author): (A) Water bath with eight fermenters and a water heating and mixing system; (B) Schematic connection of the fermenter with the cylinder holding the gas and the expansion tank. (C) system view.

The microbial degradation of soaps under anaerobic conditions was tested using an automatic fermentation system (Figure 3), which was constructed in the Department of Experimental Biology (Section of Microbiology) at Masaryk University in accordance with the VDI 4630 standard. It is a system with a steel fermenter and a glass insert with a total volume of 5 L. There were a total of eight fermenters in the system, each of which had its own outlet for measuring the biogas composition. The principle of the method is to use a mixed culture of anaerobic sludge to break down a solid substrate in the form of soap from edible oil, producing biogas as the end product. The biogas produced was measured daily based on the displacement capacity of the salt‐saturated solution in a glass cylinder above the fermenter. The resulting biogas pushed the solution out of the glass cylinder into the expansion vessel, where the value was read on the scale of the measuring cylinder. This 10‐L cylinder was filled with a mixture of water, 10 mL H_2_SO_4_ and 10 mL methyl orange, which served as a level indicator. In accordance with the VDI 4630 standard, 300 g of NaCl was used as a saturated acid solution, which served as a barrier to prevent the released gas from dissolving in this liquid (Figure 3). The biogas collected in the glass bottle could be analysed thanks to the collection point with coupling that all glass bottles were equipped with. The composition of the biogas produced was analysed using a Dräger X‐am 5600 analyser (Dräger, Lübeck, Germany). The resulting biogas yield was converted to normal conditions (*p* = 101,325 Pa; T = 273.15 K) and expressed in Nm^3^ per kg of added organic matter; the inoculum biogas yield was subtracted.

On the first day of the experiment, the sludge inoculum from the anaerobic stabilisation of the Brno–Modřice WWTP was dosed into all eight fermenters (Table 2). Two digesters in each experiment were used as a control to measure the endogenous biogas yield of the inoculum. In the remaining six fermenters, a solid sample was prepared and added to 3000 mL of anaerobic sludge. The dosage of soaps differed for each sample due to the limited amount of soap. For palm oil, the weight was 9.4 ± 0.02 g; for sunflower oil, 3.50 ± 0.01 g and for rapeseed oil, 3.52 ± 0.02 g. All fermenters were sealed with a gas‐tight lid and connected to a hose with an outlet to a glass cylinder to measure the volume of biogas produced. The steel fermenter was placed in a water bath, which was kept at a constant temperature of 40°C ± 0.2°C throughout the cultivation period using an electric heater. The retention time of the substrate in the fermenter was 21 days, and the test was terminated when the daily methane production reached more than 1% of the accumulated methane volume for three consecutive days. Anaerobic conditions prevailed in the fermenters throughout the test.

**TABLE 2:** Anaerobic soap degradation measurement parameters.

Parametr	Value
Inoculum	Sludge from anaerobic stabilisation, wastewater treatment plant; Brno–Modřice, Czech Republic; Temperature conditions mesophilic (40°C)
Tested sample	0S, 2S, 5S 0P, 3P, 4P 0R, 2R, 5R
Fermenter volume	Total volume 5 dm^3^; Sludge volume 3 dm^3^
Temperature [°C];	40°C ± 0.2°C;
Heating method	Water bath
Mixing	Manual, daily
Residence time (days)	21
Biogas yield measurement method	Based on the standard VDI 4630
Biogas composition measurement method	Gas analyser Dräger X‐am 5600; Infrared sensors for CH_4_ and CO_2_; Mixture of gases (60% CH_4_/40% CO_2_) used as calibration

### 2.8 Software

The Achat OC V320 software programme from WTW Xylem Analytics with an A K 540/B connection cable between the OxiTop OC 100 controller and the computer was used to process the output data from the OxiTop device. The data were then downloaded into an MS Excel spreadsheet. The DeODorizer programme in Python was used to calculate the growth parameters.

## 3 Results

### 3.1 Aerobic Degradation

In total, eight samplings of activated sludge were carried out within 4 months. Three samples of the soap were tested in a total of 143 bottles, with the samples labelled 0P, 1P and 2P being repeated due to low BOD values. An average value of dry matter content in activated sludge of 0.57% ± 0.04% and loss on annealing of 52.32% ± 6.93% was obtained (Table S2).

#### 3.1.1 Palm Oil Soap

**FIGURE 4:**
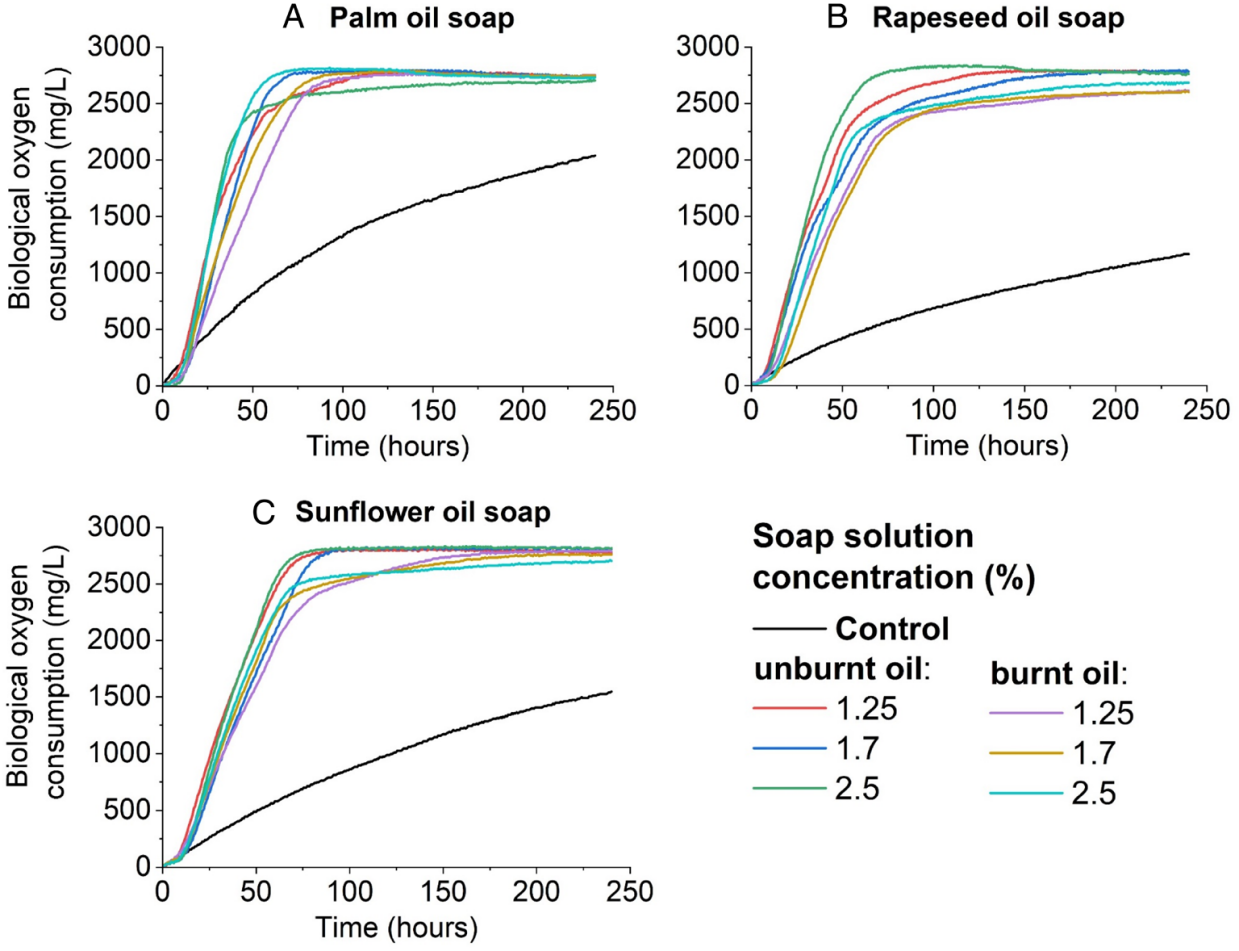
Comparison of the biological oxygen demand (BOD) of the highest and lowest degree of burning for samples: Palm oil soup (a), rapeseed oil soup (b) and sunflower oil soup (c).

After a 10‐day incubation period of activated sludge with palm oil soap in an aerobic OxiTop, typical growth curves were obtained from which a significant exponential phase between 6 and 48 h can be recognised. First, the difference between the highest and lowest degree of overburning was compared for all concentrations determined. The activated sludge with the sample with the highest degree of overburning and the lowest concentration—5P 1.25%—had the lowest growth rate, which was also confirmed by calculating the growth rate of 4.09∙10^−1^ ± 6.78∙10^−3^ h^−1^ (Figure 4a).

In comparison, the palm oil soap with the lowest overburning (at the highest concentration) 0P 2.5% showed the highest growth rate in the sludge of 6.52∙10^−1^ ± l.18∙10^−3^ h^−1^ (Table S3). However, among the 0P samples, the sample with the lowest overburning with the lowest 0P concentration of 1.25% (5.64∙10^−1^ ± 9.51∙10^−3^ h^−1^) showed a higher growth rate than the sample with a concentration of 1.7% (2.78∙10^−1^ ± 6.19∙10^−3^ h^−1^). Although the soap‐free control did not show a lag phase, no significant prolongation of this time was observed in any of the samples. The lag phase coincided identically between 6.66∙10^−1^ ± 2.22∙10^−2^ h^−1^, outside the 0P 2.5 sample (4.42 ± 1.65∙10^−1^ h^−1^). At the same time, the shortest doubling time of 1.06 ± 1.92∙10^−2^ h^−1^ was observed for this sample.

Unlike the other samples, the difference between the 3P samples is visible, especially in the longer lag phase for the 3P 2.5% and 4P 2.5% samples (Figure 1S, **A**). From the calculations, the lag phase length was 11.33 h for the 4P 2.5% sample and 10.67 h for the 3P 2.5% sample. Due to an instrument error, only one set of data was used in the graph here, and therefore it is not the average of the measurements. The 3P 1.25% sample showed the lowest growth rate among samples with this concentration, 4.05 ± 4.83∙10^−3^ h^−1^, but a shorter lag phase of 4.36 ± 7.15∙10^−2^ h^−1^.

The BOD of the control sample, only activated sludge, reached a maximum of 2041 mg/L after 240 h compared to sample 2P with a concentration of 1.7%. In sample 2P, the highest BOD concentration of 2817.5 mg/L was measured after 93 h of all tested soap samples (Figure 1B,S). The lowest measured value was measured for sample 4P 2.5% with a maximum BOD of 2795 mg/L after 232 h of cultivation. Sample 3P 2.5% had the maximum BOD 2768 mg/L after 220 h. The average BOD value of all palm oil samples was 2609.56 ± 24.37 mg/L. All growth curves are shown in Supporting Information (Figure 1C,S).

#### 3.1.2 Rapeseed Oil Soap

When comparing the lowest and the highest degree of burning for rapeseed oil, Figure 4b shows the high respiratory activity of soap made from unburnt rapeseed oil with the highest concentration. In contrast, the lowest respiration was achieved by the soap sample made from the oil with the highest overburn (5R) with a concentration of 1.7%.

Compared to soap made from palm oil, the exponential phase was prolonged up to 64 h. The soap samples again had an almost identical lag phase of 6.66∙10^−1^ ± 2.22∙10^−16^ h^−1^, except for sample 5R 2.5% (1.30 ± 1.47∙10^−1^ h^−1^) (Table S3). However, the longest maximum growth time of 8.34∙10^−1^ ± 4.37∙10^−1^ h^−1^ was observed for this sample. The 0R 1.25% sample showed the highest growth rate of 5.14∙10^−1^ ± 4.69∙10^−3^ h^−1^ with the shortest cell doubling time of 1.35 ± 1.22∙10^−2^ h,^−1^ similar to soap made from palm oil. The slowest growth rate was observed for sample 5R 1.7%, as can be seen from Figure 4b (2.03∙10^−1^ ± 4.93∙10^−3^ h^−1^). By comparing all concentrations between all degrees of burnout (Figure 2S), the slowest growth rate is observed for sample 1R 1.25% (C). Slowdown occurred within 70 h, but the calculated doubling time of 1.35∙10^−1^ ± 2.42∙10^−3^ h^−1^ was not significantly different from the 0R 1.25% sample. The average highest measured BOD value for soap made from rapeseed oil was 2714.72 ± 86.18 mg/L. The control with activated sludge without the addition of soap respired with a maximum BOD concentration of 1419.50 mg/L in 240 h, before the end of the experiment, lower than the control used with palm oil soap. At the same time, it is approximately half the value of the maximum concentration of BOD measured for comparison in the 0R sample with a concentration of 2.5%. After less than 109 h, 2839 mg/L was recorded. In the supplemental material are all the curves for the rapeseed oil sample (Figure 2S).

#### 3.1.3 Sunflower Oil Soap

When comparing the lowest and the highest degree of overburning of sunflower oil soap, the same lag phase of 6.66∙10^−1^ ± 2.22∙10^−16^ h^−1^ was calculated for all concentrations of both overburnings (Figure 4c). An equally long lag phase is also visible in the case of other overburning (Figure 3S). The exponential phase was extended to 64 h, similar to the case of soap made from palm oil. For the smallest burnout 0S, the growth rate did not differ significantly for any concentration (4.48∙10^−1^ ± 1.95∙10^−2^; 4.83 ± 8.85∙10^−2^; 4.37∙10^−1^ ± 4.77∙10^−3^ h^−1^). Compared to 5S samples, where in the case of the highest concentration of 2.5%, the maximum growth rate was 9.17∙10^−1^ ± 3.17∙10^−2^ h^−1^, and in the sample with the lowest 5S concentration of 1.25%, the maximum growth rate was the lowest 1.46 ± 3.06∙10^−2^ h^−1^ (Table S3). The visible shift in the curve is 1.25% for sample 4S (Figure 3S), whose maximum growth rate reached 3.19∙10^−1^ ± 4.35∙10^−3^ h^−1^ and the doubling time was 2.17 ± 2.96∙10^−2^ h^−1^. Similar to sample 1R 1.25%, there was a decrease in growth around 70 h.

Regarding BOD concentration, the average highest value was 2748.78 ± 52.37 mg/L for sunflower oil soap. For the 0S 1.25% sample, a BOD value of 2740 mg/L was measured already after 75 h and the maximum BOD value of 2810.5 mg/L after 108 h. In contrast, the mentioned 4S 1.25% sample only reached a BOD concentration of 1977 mg/L at the same time (75 h) and its maximum measured BOD concentration was 2718.5 mg/L after 238 h. The soap‐free control showed a maximum respiratory activity of 1546.5 mg/L. All curves of the obtained measurements are presented in Figure 3S.

Growth parameters for the lowest and highest degree of overburning of soaps P, R, S calculated using the DeODorizer programme (Table S3).

### 3.2 Anaerobic Degradations

To compare the ability of the microbial community in the anaerobic sludge to degrade soaps produced from edible oil, three types of soaps were used as in the aerobic experiment. The methane production data were obtained by measurements comparing three different degradation processes for each soap. The average dry matter value of the anaerobic sludge was determined to be 3.36% ± 0.11% and the average loss on annealing was 60.53% ± 0.87% (Table S4).

#### 3.2.1 Palm Oil Soap

**FIGURE 5:**
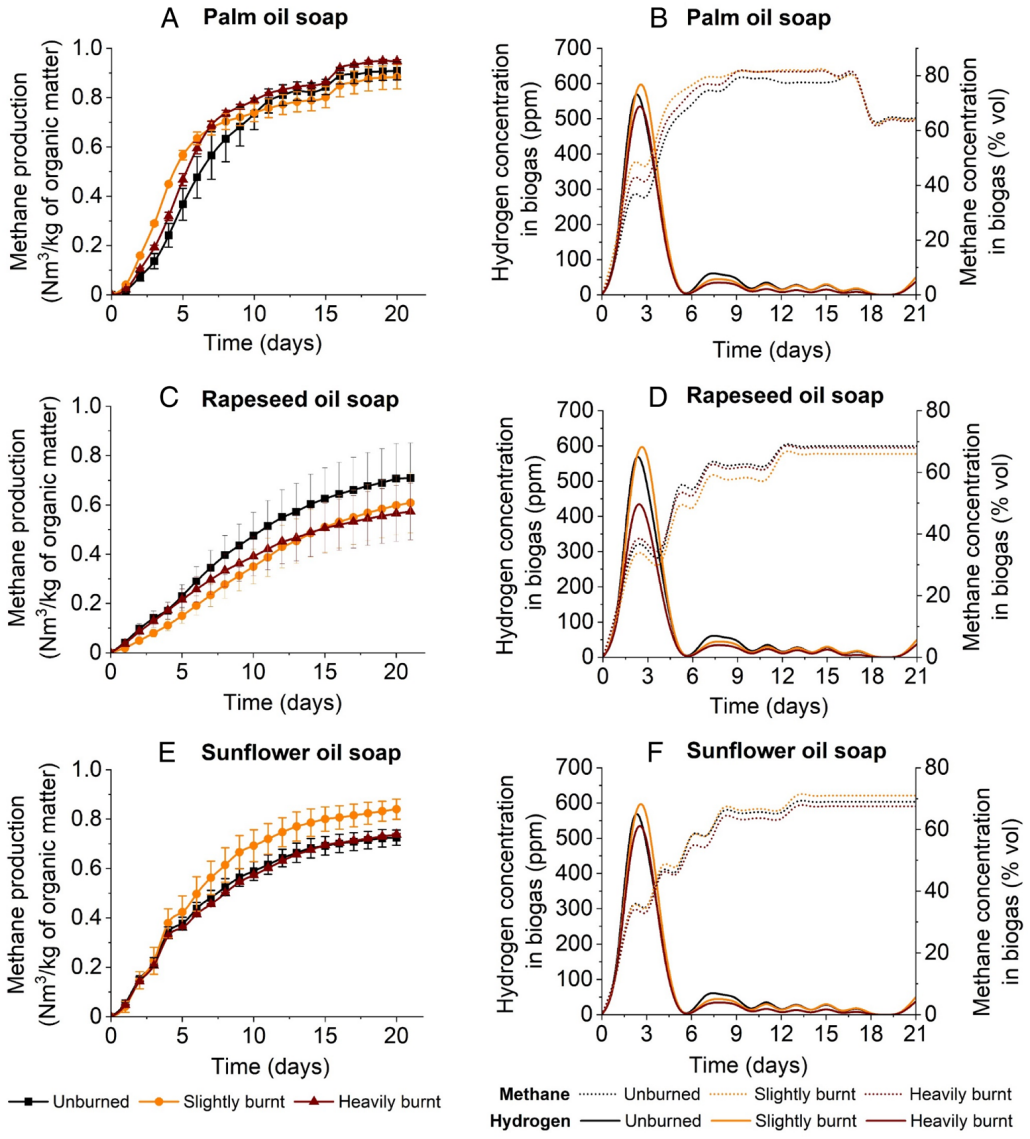
Cumulative methane yield (a, c, e) and hydrogen in biogas (b, d, f) using oil soap samples.

On the cumulative methane production curve for soaps made from palm oil, the development of the curves for the individual degrees of overburning is the same, but different compared to soaps made from rapeseed and sunflower oil (Figure 5a). The highest methane production occurred between the 3rd and 14th day when it reached 0.8640 Nm^3^/kg_ODM_ specifically for sample 4P. and then increased again to a total of 0.9616 Nm^3^/kg_ODM_ between the 14th and 21st day. The lowest total methane production was observed in sample 3P. There was no significant lag phase.

In the soap made from palm oil, an increase in the hydrogen concentration was observed at the beginning of the experiment. Most of the hydrogen was produced on the second day in sample 0P, totalling 662.5 ppm (Figure 5b). In sample 3P, hydrogen production was highest on day 3 with a total of 637.5 ppm, and in sample 4P also on day 3 with 555 ppm. Methane production increased sharply between days 2 and 7, and a significant decrease in methane production was observed between days 17 and 18. The methane concentration in the biogas in samples 3P and 4P was up to 81.5 vol% of the biogas, and in sample 0P around 77.5 vol%. On the fifth day, residual oxygen was also present.

#### 3.2.2 Rapeseed Oil Soap

The cumulative increase in methane was lower in the soaps made from rapeseed oil than in the soaps made from palm oil. The increase was gradual over the 21 days and peaked in the sample with the lowest degree of burning (sample 0R). Here, there was not even a significant difference between the average values of the two fermenters, which both showed the same increase with an average standard deviation of ±0.01, as shown in (Figure 5c). The average methane production in six fermenters was determined to be 0.76 ± 0.07 Nm^3^/kg_ODM_. The total methane production for each sample was as follows: 0R 0.7683 Nm^3^/kg_ODM_; 2R 0.7810 Nm^3^/kg_ODM_ and 5R 0.7327 Nm^3^/kg_ODM_ (Figure 5d). In the soap produced from rapeseed oil, we can therefore observe a slight effect of the highest degree of burning on the reduced methane production. The anaerobic sludge without soap addition was used as a control and its total biogas yield was 0.0085 Nm^3^/kg_ODM_. The total methane production in the control was 0.0039 Nm^3^/kg_ODM_.

**FIGURE 6:**
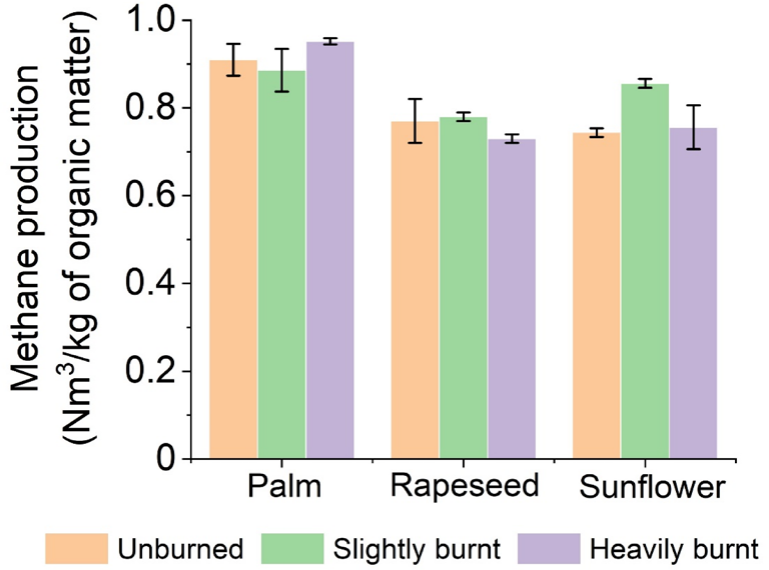
Comparison of methane production after 21 days of fermentation of soaps made from different burnt oils.

Analogous to the case of soap produced from sunflower oil, a diagram was drawn up comparing the production of biogas and methane (Figure 6). In sample 0R, hydrogen production was highest on day 2 at 662 ppm and then decreased. For sample 2R, production was highest on day 3 at 637.5 ppm. Moderate hydrogen production was also recorded during the period when methane already dominated. Methane was produced most rapidly during the first 7 days and then levelled off, similar to the soap produced from sunflower oil, but at a higher rate around 68.5%_vol_.

#### 3.2.3 Sunflower Oil Soap

The highest biogas yield was determined for soap sample 2S with 1.33 Nm^3^/kg_ODM_. An increase in hydrogen concentration in the fermenter was observed in the first 4 days. After the fifth day, the concentration dropped rapidly, while the methane concentration in the biogas increased significantly (Figure 5e).

The methane concentration increased between the 2nd and 8th day and then stabilised at around 60%_vol_ in all three samples by the 12th day. In the first 3 days, there was also a residual amount of oxygen in the fermenter before its concentration was completely depleted and anaerobic respiration prevailed. Furthermore, no hydrogen was detected in the biogas.

Figure 5f shows the cumulative production of methane produced during fermentation with a retention time of 21 days, converted into Nm^3^/kg_ODM_. Each curve represents the average of methane production from two measurements, with values read every day. No lag phase was observed, and the highest increase occurred between 4 and 12 days. The highest cumulative methane production can be seen in the 2S soap sample, which was slightly overburnt. On the contrary, the samples with the lowest and highest degrees of burnout showed an almost identical trend. However, the 5S sample does not accurately reflect the effect of the highest degree of burnout on methane production, as only one set of values was used and not an average value. In one of the fermenters, gas was released due to poor sealing of the system, so the data here cannot be compared with the accuracy of the standard deviation.

During 21 days, an average of 0.79 ± 0.23 Nm^3^/kg_ODM_ methane was produced in each of the six fermenters used with the solid soap sample. If we compare the production of each soap sample in Figure 5, we see the highest production in sample 2S, whose maximum methane production was 0.8559 Nm^3^/kg_ODM_, compared to sample 0S, whose production was 0.7463 Nm^3^/kg_ODM_. For sample 5S, an average methane production of 0.7560 Nm^3^/kg_ODM_ was determined (Figure 5f). The soap‐free anaerobic sludge was used as a control, and a total methane production of 0.0017 Nm^3^/kg_ODM_ was measured.

### 3.3 Comparison of Cumulative Methane Production Between Individual Soap Samples

Three different degrees of overburning were tested for each type of soap to determine the effect of the degree of overburning on methane production during 21 days of residence in the fermenter (Figure 6). For none of these soaps, we can observe a trend that would confirm the fact that the degree of burnout has an effect on methane production. The average methane production of the three controls used was 0.0025 ± 0.001 Nm^3^/kg_ODM_. In comparison, the overall average methane production of all samples was 0.8286 ± 0.27 Nm^3^/kg_ODM_.

The highest production of methane was achieved in samples of soap made from palm oil: 0.9095 (0P); 0.8859 (3P) and the most of them (4P) 0.9516 Nm^3^/kg_ODM_. Here, the fourth degree was used as the highest burning degree due to the limited amount of soap samples. For soap samples made from sunflower and rapeseed oil, the values were up to 0.9 Nm^3^/kg_ODM_. In the case of soaps made from sunflower oil, sample (2S) had the highest yield of 0.8559 Nm^3^/kg_ODM_. Also, in the case of soap produced from rapeseed oil, the middle degree of overburning had the highest methane production of 0.7810 Nm^3^/kg_ODM_.

## 4 Discussion

During frying, substances are formed that are more polar than the triacylglycerols of the lipids. The CPH value indicates the degree of oil contamination by these substances compared to the original state (Antonic et al. 2021). As a result of the increased production of oil crops (FAO 2021), a considerable amount of waste fats, oils, greases and waxes is also produced. In industrialised countries, the annual production of waste oil is up to 50 kg per person; in less developed countries, this production is 20 kg per person. In recent years, there have been a significant number of cases where this waste has accumulated in the sewerage system and completely blocked it (Wallace et al. 2017). In London alone, up to 10,000 kg of so‐called fatbergs accumulate every year, and in 2017 a record was set when 1301 fatbergs (250 m long) blocked the pipes in the Whitechapel district. The extracted fat was then used to produce 10,000 L of biodiesel (Frangoul 2017).

Another problem caused by burnt fats is the most common peroxidation product—malondialdehydes (MDA). These toxic substances are mainly produced during food frying, and their consumption increases susceptibility to cardiovascular disease, liver problems and cancer (Ma et al. 2021). Human health can also be affected within the food chain by the consumption of fish and other aquatic animals that come into contact with harmful oil intermediates in the aquatic environment (Foo et al. 2021).

The removal of fats and oils during biological wastewater treatment has been investigated in several studies. For example, the study by Liu et al. (2004) found that fats and oils with a concentration of up to 600 mg/L can be degraded in activated sludge with an efficiency of 90% (at a temperature of 21°C and a retention time of 5 days). At higher fat concentrations, a significant decrease in the chemical oxygen demand (COD) was observed (Liu et al. 2004).

Currently, a lot of research is focused on the production of biosurfactants, that is, biological surface‐active substances that could improve cleaning efficiency. One of the most potentially promising biosurfactants is rhamnolipid, which is produced by the bacterium *Pseudomonas aeruginosa* . Using different concentrations of rhamnolipid between 22.5 mg/L and 90 mg/L, up to 93% of all fats in activated sludge were degraded in 30 h (Zhang et al. 2009). In this bacterium, it was also found that the production of rhamnolipid can be greatly enhanced when cooking waste oil is used as a carbon source during cultivation. However, this application is greatly limited by the production price, which is approximately 2600 €/m^3^ of rhamnolipid (Zhu et al. 2007).

Soap production has been proposed as an ecological and cheap alternative to the disposal of used cooking oil. The world's leading supplier of soap is Poland, which earned 231 million euros from soap production in 2021. Despite this high amount, the price of a soap product on the market fluctuates between €1.5 and €10 (CBI 2023). Taking into account the price of ingredients and production energy, the production of 65 soaps from cooking oil as input material was estimated at €5.60, that is, only €0.09 per soap (Félix et al. 2017).

In the biological process of wastewater treatment, it has already been proven in the past that saponified fat is broken down up to four times faster than crude fat. Furthermore, in the case of crude fat, solid fat aggregates were trapped around the aeration system and were therefore not available for the biomass (Lefebvre et al. 1998).

It is often discussed in the literature that foam forms in wastewater treatment plants with a high concentration of cleaning agents, the formation of which is additionally supported by massive aeration in the activation tank. The foam layer on the surface reduces the ability of oxygen to penetrate the water, which leads to a reduction in microbial respiration and purification performance by 30% to 40% (Mousavi and Khodadoost 2019). Foaming has also been linked to serious operational failures of wastewater treatment plants—particularly pump failures, metre failures and scaling on tank walls. In anaerobic technologies, pipe fouling can occur, which can increase the pressure in the bioreactors. As a result of these problems, the bioreactor can become unstable, its volume, retention time and the degree of substrate degradation decrease, which has an impact on the reduction of biogas yield (Moeller et al. 2012).

However, this reasoning could not be confirmed when using saponification products from edible oils due to the measured values of biological oxygen consumption with the OxiTop device at all soap concentrations used and also during anaerobic degradation with measured biogas yield. At the same time, no massive foaming was observed during the production of the soap solutions, which indicates the possible use of these soaps. Foaming could possibly be reduced in the activation tank by discontinuous aeration, as suggested by Lefebvre et al. (1998). The formation of incrustations during aerobic or anaerobic degradation of soap samples produced from waste oils was also not observed.

### 4.1 Aerobic Degradation

By comparing the BOD of all soap types with a control without added soap, it was confirmed that soap made from used oils with different CPH content did not inhibit microbial respiration. The lag phase was within 6 h for most samples, in contrast to the study in which the biodegradation of matolin was investigated. Here, a lag phase of 10 to 40 h was observed. The influence of soaps on the respiration activity of sewage sludge is therefore lower compared to this product (Tauferova et al. 2021).

In the literature, the degradation of palmitic and stearic acid in activated sludge is often reported to be up to five times slower than that of unsaturated fatty acids (Novak and Kraus 1973). Palmitic acid is the main component of palm oil, and this may partly explain the different results compared to other soaps made from sunflower and rapeseed oil. Here, a longer lag phase was observed for some grades of burnout. The basic chemical parameters of the activated sludge were determined: an average pH of 7.27 ± 0.35; redox potential 158.75 ± 12.92 mV; conductivity 1218.38 ± 97.01 mS/cm. The pH value was slightly increased compared to the values reported in the literature but corresponded to the range of 6–7 (Anjum et al. 2016). The selected 10‐day residence time in the system proved to be optimal, since the exponential phase of the growth of microorganisms in the sludge took place mostly between 6 and 48 h.

The aim of this work was not to analyse the microbial composition of the sludge. Each sampling took place at a different time and at different seasons when different environmental conditions affect the wastewater treatment plants. The dynamics of the composition can therefore be different, especially in connection with low temperatures in the winter and spring months. The relative abundance of the genus Zooglea (most abundant in autumn and least abundant in spring), which plays an important role in floc formation (Johnston and Behrens 2020), could also contribute to the differences. However, as shown in a study on the global composition of activated sludge, the basic composition of sludge is generally represented by 28 taxa, which form the basic functional core for ensuring effective wastewater treatment (Wu et al. 2019).

### 4.2 Anaerobic Degradation

In the case of this study, soaps were produced by saponification using NaOH as a strong base for fat hydrolysis. In another study, the influence of calcium ions used for the degradation of soap from rapeseed oil and the production of methane was tested. Three different bases were used as saponification agents: CaCl_2_, CaSO_4_ and Ca(OH)_2_, and anaerobic sludge from the UASB reactor was used as inoculum. Although the total value of methane production cannot be compared here due to the longer residence time (40 days), the cumulative methane production after 21 days showed the same trend as in our case, when methane production reached 0.9516 Nm^3^/kg_ODM_, and at the same time there was no lag phase. The highest methane yield was recorded using Ca(OH)_2_ as a saponification agent (Wu et al. 2022).

In all cases of soaps produced from edible oils, the total amount of biogas produced was compared with the main component, methane. Methanogenesis is undoubtedly one of the most important metabolic processes occurring in anaerobic sludge, as has been demonstrated in a number of studies (Stams et al. 2012). The typical residence time for anaerobic sludge stabilisation is between 18 and 30 days, and methanogenesis occurred around day 3, which corresponds to mesophilic conditions in the digester (40°C ± 0.2°C), as tested for cumulative methane production at different temperatures (Vítěz et al. 2020).

Other anaerobic sludge conditions were adjusted to a pH of 7.38 ± 0.02, which is slightly higher than aerobic sludge, but at the same time corresponds to the values reported in the literature for anaerobic sludge in the range of 6.5–7.5, which is suitable for acetogenesis and methanogenesis (Liu et al. 2008). Alternatively, the process could also be made more efficient by lowering the pH value. The organic matter content determined by annealing was 60.53% ± 0.87% and the dry matter content was 3.36% ± 0.11%.

The redox potential was negative (−282.67 ± 47.62 mV), which corresponds to anaerobic redox conditions. During the process of anaerobic stabilisation, the redox potential can fluctuate significantly between −200 and −500 mV (Bartkowska 2014).

The conductivity was 7.61 ± 0.51 mS/cm compared to the activated sludge, which is significantly lower, due to the higher density of the sludge.

The total methane production was highest for soaps made from palm oil compared to soaps made from rapeseed and sunflower oil. This fact could be explained by the different composition of the fatty acids, with palm oil having a higher proportion of saturated fatty acids and at the same time containing no trans fats.

Decomposition is therefore slower (Absalome et al. 2020; Antonic et al. 2021; Orsavova et al. 2015). At the same time, the MDA content may also play a role, as palm oil contains less MDA: 0.45 μg/g (P1) to 0.94 μg/g (P5), compared to the other soap samples. The MDA content of soap made from rapeseed oil was 2.58 μg/g (R1) and 4.46 μg/g (S1) for soap made from sunflower oil (Antonic et al. 2021).

Biogas produced from a fat‐rich substrate clearly has a higher proportion of methane in the biogas than biogas produced from hydrocarbons and proteins. Volatiles and ammonia are the main intermediates formed during anaerobic digestion, and a number of studies have mentioned potential toxicity in case of excessive accumulation of these substances during decomposition (Dasa et al. 2016). This fact confirms that the toxic effect can be avoided with the help of saponification. During the hydrolysis of lipids, all fatty acids are degraded so that their negative accumulation does not occur during anaerobic fermentation. Compared to a study comparing the effect of palm oil on anaerobic sludge (Dasa et al. 2016), increased methane production was demonstrated. Significant degradation of soap and support of methanogenesis in anaerobic sludge was confirmed compared to controls for all types of soaps.

Co‐fermentation of sewage sludge with kitchen waste has often been proposed in Europe, but has not yet been put into practice due to its solution. Kitchen waste is a variable waste that differs in its composition (Iacovidou et al. 2012). It is therefore important to separate and recycle this waste according to its individual components.

## 5 Conclusions

Cooking oils are an important part of every household and it is impossible to imagine life without them. At the same time, however, they are also associated with negative effects on human health and the environment. Burnt fat in particular is a material that is not normally recycled. For this reason, a large amount of it ends up in the sewage system and in wastewater treatment plants. The impetus for this work was a study on the utilisation of cooking oil in the form of soap, in which the physicochemical parameters of its composition were investigated. It has been shown that the production costs of soaps are relatively low, considering the costs that can be incurred when removing fats from the sewerage system.

A total of three types of the most commonly used oils for soap production were tested to see if they would have different cleaning capabilities. According to the calculated growth parameters, no influence of the soaps on the length of the lag phase was found. The microorganisms were able to adapt quickly to the environmental conditions and degraded long fatty acid chains under both aerobic and anaerobic conditions. The growth rate was found to be different, but this had no effect on the overall biological production of oxygen. Although the soaps were tested at different levels of scorch and the soap solutions were prepared at different concentrations, no differential effect of these factors on microbial respiration was observed over a 10‐day period. This was also not the case when measuring the cumulative methane production during a 21‐day retention period. The conversion of soaps into biogas by anaerobic microorganisms led to successful results. Methane was detected as the dominant component of the biogas, which predominated in the digesters from day 3 of fermentation. Hydrogen was also detected to a lesser extent, confirming the syntrophic cooperation of the microorganisms in the system.

The most important finding of this work is the elimination of the toxic effect of the saponification products on the sewage sludge, which opens up the possibility of incorporating them into the wastewater treatment process without any problems. It was shown that they can improve the efficiency of the treatment by increasing the respiration activity of the activated sludge. At the same time, it was shown that soaps can serve as a substrate for methanogenic microorganisms to produce methane and hydrogen in biogas components.

The study presents an innovative approach to recycle burnt cooking oils into soaps that can be later processed without problems in wastewater treatment processes, enhancing microbial respiration and biogas yield without toxic effects on sewage sludge. This dual benefit of environmental sustainability and economic efficiency offers a promising solution for managing used cooking oils and improving the operation of wastewater treatment plants.

## Author Contributions

Dani Dordevic, Monika Vítězová, Tomáš Vítěz, Simona Dordevic, Monika Hamšíková: conceptualisation, methodology, data curation, formal analysis, writing – original draft; Dani Dordevic, Monika Vítězová, Tomáš Vítěz, Ivan Kushkevych: data curation, formal analysis; Dani Dordevic, Monika Vítězová, Tomáš Vítěz: investigation, resources; Dani Dordevic, Monika Vítězová, Tomáš Vítěz and Ivan Kushkevych: conceptualisation, funding acquisition, resources, validation, visualisation, writing – review and editing; Ivan Kushkevych: visualisation, supervision.

## Conflicts of Interest

The authors declare no conflicts of interest.

## Supporting information


**Data S1.** Supporting Information.

## Data Availability

The data that support the findings of this study are available on request from the corresponding author. The data are not publicly available due to privacy or ethical restrictions.
