# Supplementary material for: Enhancing Wastewater Treatment Efficiency: Utilising Saponification Products for Sustainable Cleaning Processes
Source: Environ Microbiol Rep. 2025 Jul 9;17(4):e70124. doi: 10.1111/1758-2229.70124 (PMC12239153; doi:10.1111/1758-2229.70124)
Supplement: Supplementary file 1 — Data S1. Supporting Information. [file EMI4-17-e70124-s001.docx]

**Supplemental material**

**Table 1S**. BOD experiment setup, sludge volume, mineral medium volume, soap volume

| Bottle number | Volume of washed activated sludge [mL] | The volume of the mineral medium according to OECD [mL] | Volume of soap solution [mL] | The degree of burning with the concentration of the soap solution (%) |
| --- | --- | --- | --- | --- |
| **1; 2** | 15 | 23.5 | 5 | 0P 2.5 |
| **3, 4** | 15 | 23.5 | 5 | 0P 1.7 |
| **5, 6** | 15 | 23.5 | 5 | 0P 1.25 |
| **7, 8** | 15 | 23.5 | 5 | 1P 2.5 |
| **9, 13** | 15 | 23.5 | 5 | 1P 1.7 |
| **11, 12** | 15 | 23.5 | 5 | 1P 1.25 |
| **13, 14** | 15 | 23.5 | 5 | 2P 2.5 |
| **15, 16** | 15 | 23.5 | 5 | 2P 1.7 |
| **17, 18** | 15 | 23.5 | 5 | 2P 1.25 |
| **14, 15** | 15 | 28.5 | 0 | Control |
| **10** | 15 | 28.5 | 0 | Control |

**Table 2S**. Parameters of activated sludge

| Sample number | Date collection | Place collection | Sample description | Group | pH | NL [g/L] | Redox potential [mV] | Conductivity [μS/cm] | Dry Matter [%] | Organic Dry Matter [%_DM_] |
| --- | --- | --- | --- | --- | --- | --- | --- | --- | --- | --- |
| 220001MH | 28.02.2022 | WWTP Modřice | sludge, activation tank | liquid | 7.00 | 3.70 | 150 | 1440 | 0.66±0.01 | 66.14±0.41 |
| 220002MH | 14.03.2022 | WWTP Modřice | sludge, activation tank | liquid | 7.20 | 3.70 | 170 | 1220 | 0.57±0.01 | 88.08±0.12 |
| 220003MH | 28.03.2022 | WWTP Modřice | sludge, activation tank | liquid | 7.05 | 3.60 | 132 | 1213 | 0.65±0.12 | 75.48±1.39 |
| 220004MH | 14.04.2022 | WWTP Modřice | sludge, activation tank | liquid | 7.10 | 3.70 | 168 | 1219 | 0.48±0.01 | 68.66±0.73 |
| 220005MH | 25.04.2022 | WWTP Modřice | sludge, activation tank | liquid | 8.14 | 3.70 | 160 | 1172 | 0.51±0.01 | 69.20±0.22 |
| 220006MH | 10.05.2022 | WWTP Modřice | sludge, activation tank | liquid | 7.14 | 3.70 | 170 | 1150 | 0.64±0.46 | 72.73±28.74 |
| 220007MH | 26.05.2022 | WWTP Modřice | sludge, activation tank | liquid | 7.39 | 3.70 | 150 | 1250 | 0.38±0.00 | 72.06±1.62 |
| 220008MH | 14.06.2022 | WWTP Modřice | sludge, activation tank | liquid | 7.14 | 3.70 | 170 | 1083 | 0.68±0.08 | 70.39±1.68 |

WWTP – wastewater treatment plant; ± standard deviation

**Table 3S**. Determination of kinetics parameters

| Sample | Growth Rate [h^-1^] | Maximum Growth  Rate [h^-1^] | Doubling time  [h] | Lag phase  [h] |
| --- | --- | --- | --- | --- |
| **0P 2.5 %** | 6.52∙10^-1^±1.18∙10^-3^ | 7.89∙10^-1^±2.44∙10^-1^ | 1.06±1,92∙10^-2^ | 4.42±1.65∙10^-1^ |
| **0P 1.7 %** | 2.78∙10^-1^ ± 6,19∙10^-3^ | 6.66∙10^-1^±2.22∙10^-16^ | 2.49±5.57∙10^-2^ | 6.66∙10^-1^±2.22∙10^-16^ |
| **0P 1.25 %** | 5.64∙10^-1^ ± 9.51∙10^-3^ | 6.66∙10^-1^±2.22∙10^-16^ | 1.22±2.07∙10^-2^ | 6.66∙10^-1^±2.22∙10^-16^ |
| **5P 2.5 %** | 5.67∙10^-1^ ± 1.42∙10^-2^ | 6.66∙10^-1^±2.22∙10^-16^ | 1.22±3.07∙10^-2^ | 6.66∙10^-1^±2.22∙10^-16^ |
| **5P 1.7 %** | 4.09∙10^-1^ ± 4.75∙10^-3^ | 6.66∙10^-1^±2.22∙10^-16^ | 1.69±1.96∙10^-2^ | 6.66∙10^-1^±2.22∙10^-16^ |
| **5P 1.25 %** | 4.09∙10^-1^ ± 6.78∙10^-3^ | 6.66∙10^-1^±2.22∙10^-16^ | 1.69±2.81∙10^-2^ | 6.66∙10^-1^±2.22∙10^-16^ |
| **0R 2.5 %** | 3.22∙10^-1^ ± 8.56∙10^-3^ | 6.66∙10^-1^±2.22∙10^-16^ | 2.15±5.73∙10^-2^ | 6.66∙10^-1^±2.22∙10^-16^ |
| **0R 1.7 %** | 2.04∙10^-1^ ± 4.93∙10^-3^ | 2.19∙10^-1^±1.22∙10^-16^ | 3.40±8.11∙10^-2^ | 6.73∙10^-1^±1.11∙10^-2^ |
| **0R 1.25 %** | 5.14∙10^-1^ ± 4.69∙10^-3^ | 6.66∙10^-1^±2.22∙10^-16^ | 1.35±1.22∙10^-2^ | 6.66∙10^-1^±2.22∙10^-16^ |
| **5R 2.5 %** | 2.19∙10^-1^ ± 2.31∙10^-3^ | 8.34∙10^-1^±4.37∙10^-1^ | 3.15±3.32∙10^-2^ | 1.30±1.47∙10^-1^ |
| **5R 1.7 %** | 2.03∙10^-1^ ± 4.93∙10^-3^ | 2.19∙10^-1^±1.23 | 3.40±8.11∙10^-2^ | 6.73∙10^-1^±1.11∙10^-2^ |
| **5R 1.25 %** | 5.01∙10^-1^ ± 1.09∙10^-2^ | 6.66∙10^-1^±2.22∙10^-16^ | 1.38±3.03∙10^-2^ | 6.66∙10^-1^±2.22∙10^-16^ |
| **0S 2.5 %** | 4.48∙10^-1^ ± 1.95∙10^-2^ | 6.66∙10^-1^±2.22∙10^-16^ | 1.55±6.71∙10^-2^ | 6.66∙10^-1^±2.22∙10^-16^ |
| **0S 1.7 %** | 4.83 ± 8.85∙10^-2^ | 6.66∙10^-1^±2.22∙10^-16^ | 1.43∙10^-1^±2.52∙10^-3^ | 6.66∙10^-1^±2.22∙10^-16^ |
| **0S 1.25 %** | 4.37∙10^-1^ ± 4.77∙10^-3^ | 6.66∙10^-1^±2.22∙10^-16^ | 1.58±1.73∙10^-2^ | 6.66∙10^-1^±2.22∙10^-16^ |
| **5S 2.5 %** | 9.17∙10^-1^ ± 3.17∙10^-2^ | 6.66∙10^-1^±2.22∙10^-16^ | 7.56±2.22∙10^-2^ | 6.66∙10^-1^±2.22∙10^-16^ |
| **5S 1.7 %** | 5.96∙10^-1^ ± 1.78∙10^-3^ | 6.66∙10^-1^±2.22∙10^-16^ | 1.16±3.48∙10^-2^ | 6.66∙10^-1^±2.22∙10^-16^ |
| **5S 1.25 %** | 1.46 ± 3.06∙10^-2^ | 6.66∙10^-1^±2.22∙10^-16^ | 4.73±9.82∙10^-2^ | 6.66∙10^-1^±2.22∙10^-16^ |

± standard deviation

**Table 4S**. Determination of parameters of anaerobic sludge and soap samples

| Sample number | Collection date | Place of collection | Sample description | Group | pH | Redox potential [mV] | Conductivity [mS/cm] | Dry Matter [%] | Organic Dry Matter [%_DM_] |
| --- | --- | --- | --- | --- | --- | --- | --- | --- | --- |
| 2204MH | 03.11.2022 | WWTPModřice | sludge, anaerobic stabilization | liquid | 7.40 | -248±10 | 8.34 | 3.50±0.14 | 59.34±1.60 |
| 2205MH | 03.11.2022 | – | soap 0P | solid | – | – | – | 97.39±0,01 | 84.76±0.01 |
| 2206MH | 03.11.2022 | – | soap 3P | solid | – | – | – | 98.00±0.14 | 85.33±0.03 |
| 2207MH | 03.11.2022 | – | soap 4P | solid | – | – | – | 98.11±0.03 | 85.32±0.03 |
| 2301MH | 04.01.2023 | WWTP Modřice | sludge, anaerobic stabilization | liquid | 7.36 | -250±10 | 7.60 | 3.31±0.01 | 60.81±0.09 |
| 2302MH | 04.01.2023 | – | soap 0S | solid | – | – | – | 97.26±0.05 | 85.36±0.14 |
| 2303MH | 04.01.2023 | – | soap 2S | solid | – | – | – | 97.30±0.22 | 85.55±0.02 |
| 2304MH | 04.01.2023 | – | soap 5S | solid | – | – | – | 96.85±0.12 | 85.41±0.13 |
| 2305MH | 09.02.2023 | WWTP Modřice | sludge, anaerobic stabilization | liquid | 7.38 | -350±10 | 7.09 | 3.27±0.01 | 61.43±0.05 |
| 2306MH | 09.02.2023 | – | soap 0R | solid | – | – | – | 95.96±0.22 | 86.60±0.13 |
| 2307MH | 09.02.2023 | – | soap 2R | solid | – | – | – | 95.77±0.66 | 86.59±0.17 |
| 2308MH | 09.02.2023 | – | soap 5R | solid | – | – | – | 96.93±0.02 | 86.30±0.05 |

± standard deviation


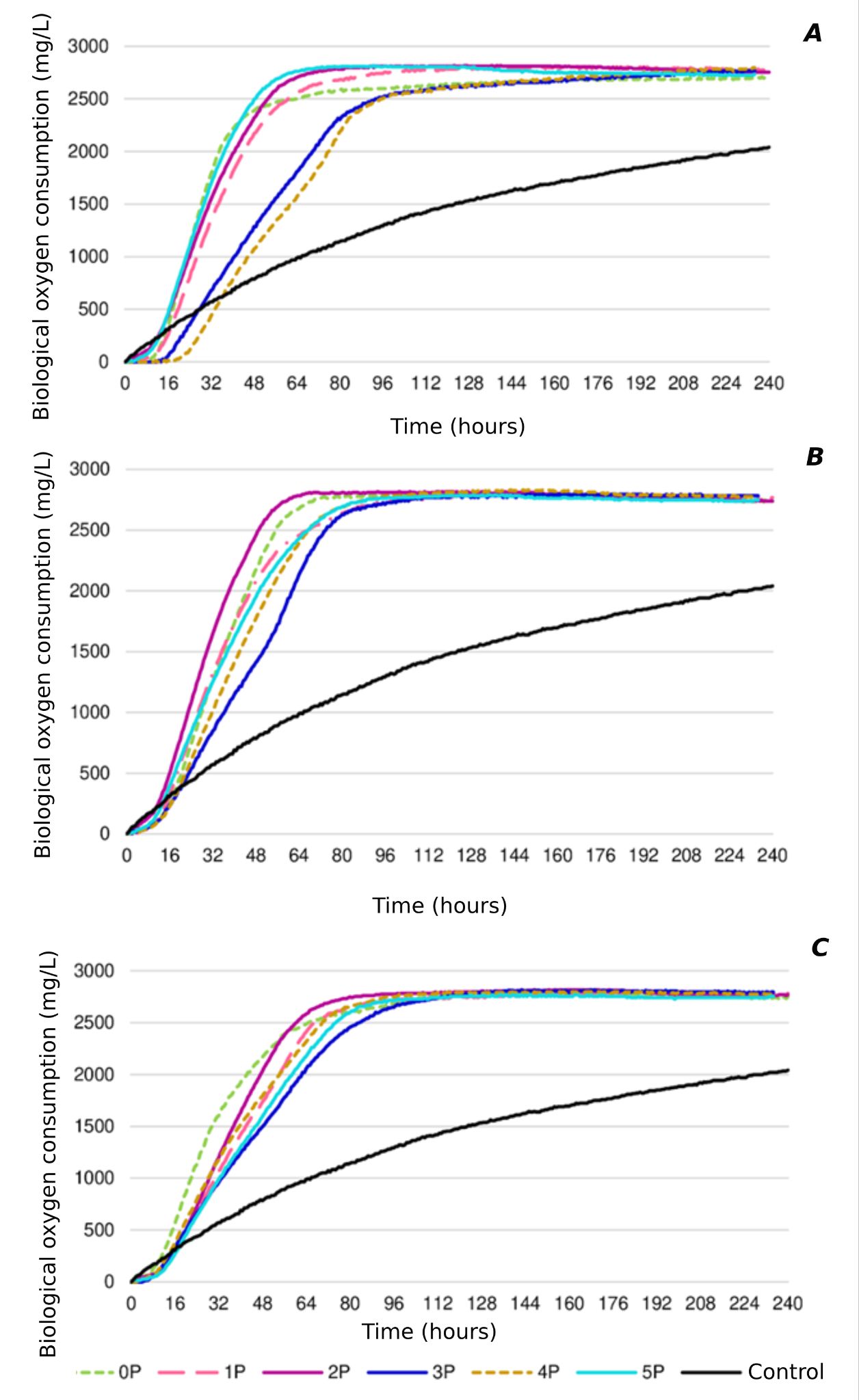


**Figure 1S**. Biological oxygen demand of activated sludge with addition of palm oil soap samples. A concentration 2.5%; B concentration 1.7%; C concentration 1.25%


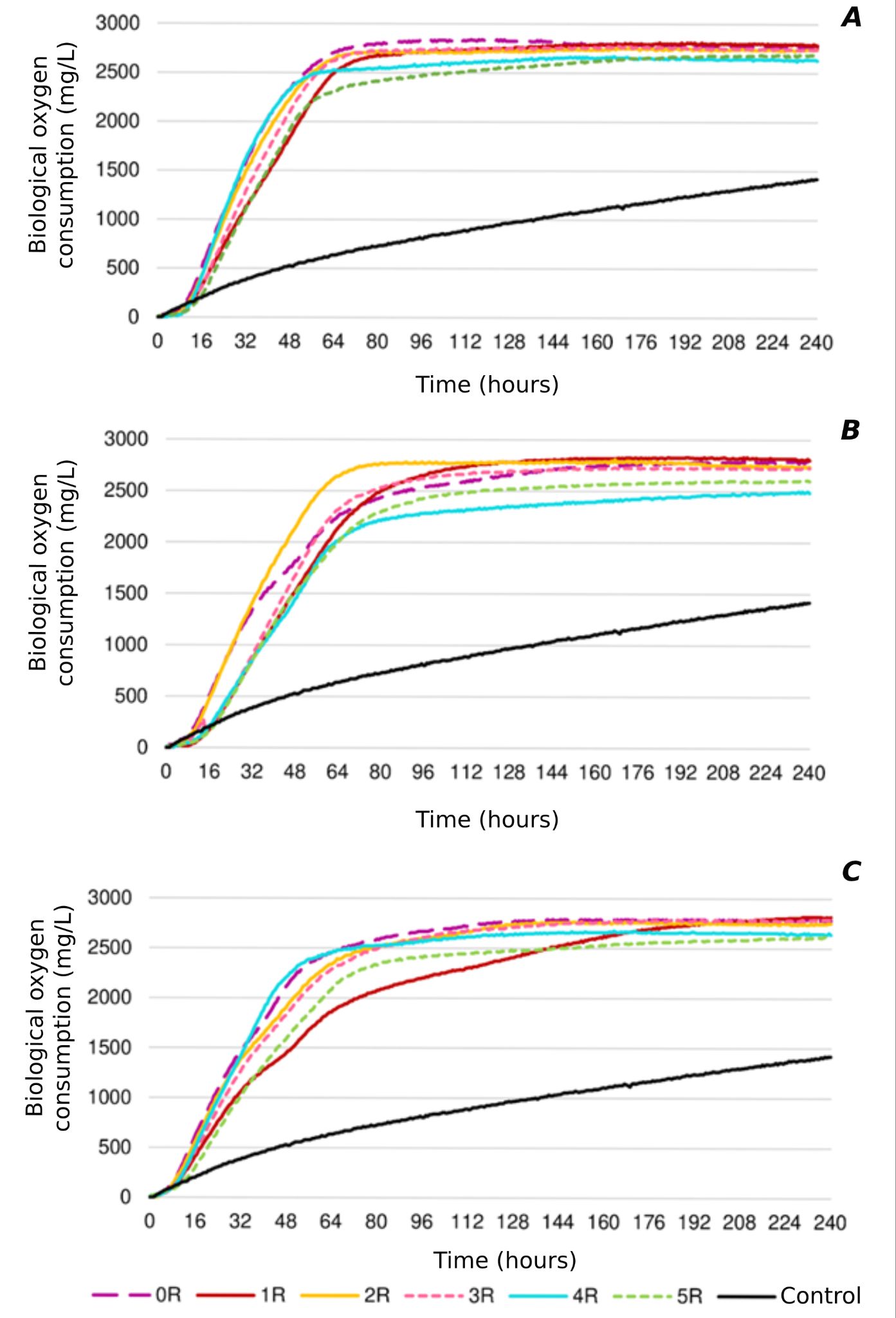


**Figure 2S**. Biological oxygen demand of activated sludge with the addition of rapeseed oil soap samples: A concentration 2.5%; B concentration 1.7%; C concentration 1.25%


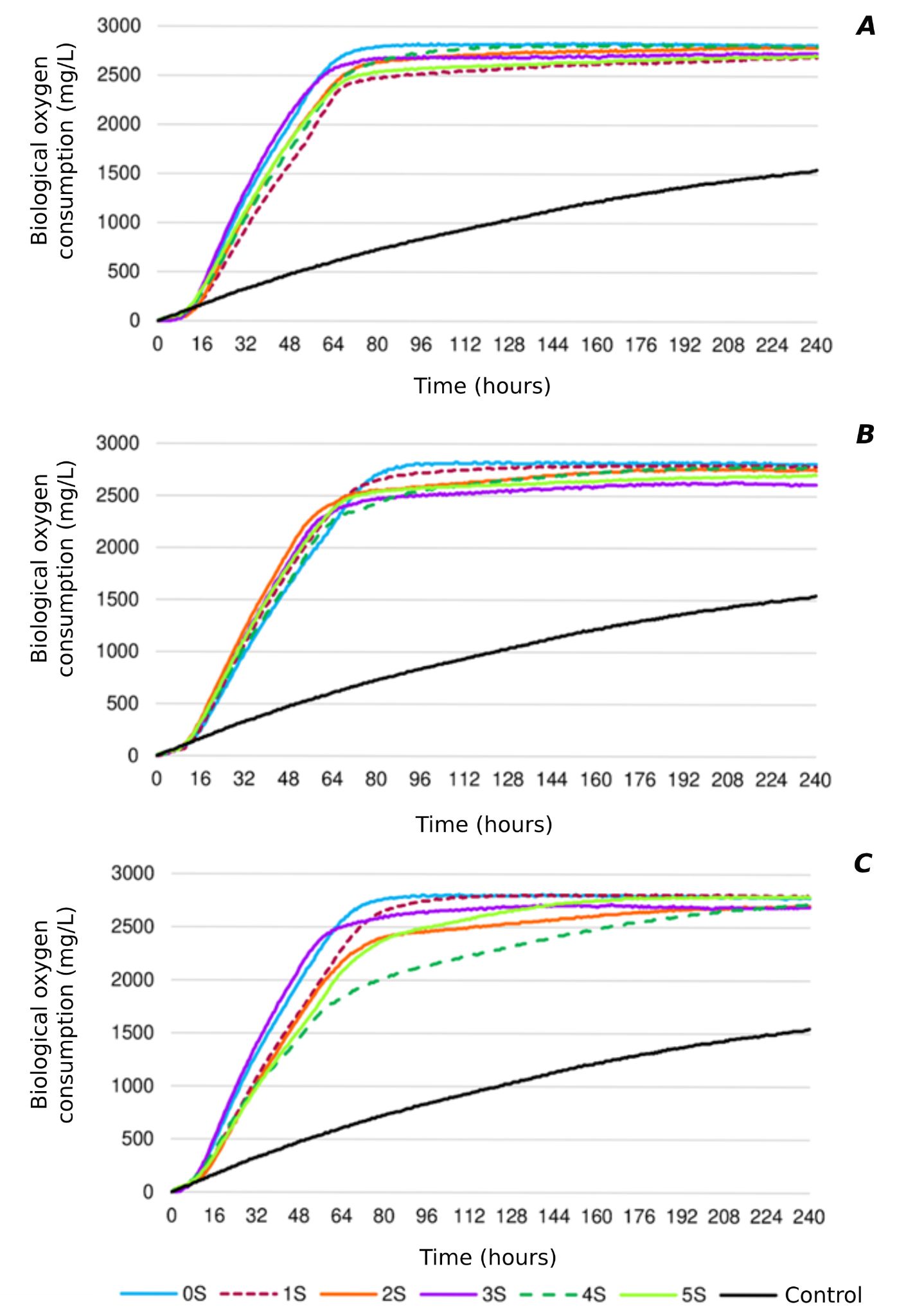


**Figure 3S.** Biological oxygen demand of activated sludge with the addition of sunflower oil soap samples: A concentration of 2.5%; B concentration 1.7%; C concentration 1.25%
